# Female urethral diverticular carcinoma: a case report and review of the literature

**DOI:** 10.3389/fonc.2024.1503116

**Published:** 2025-01-27

**Authors:** Dan Li, Fan Hua Meng, Xu Yang

**Affiliations:** ^1^ Gynecology Department, Chengdu Fifth People’s Hospital, Chengdu, China; ^2^ Urology Department, The First Hospital of Fuyang District, Hangzhou, China

**Keywords:** cancer, diverticulum, urethra, clinical manifestations, treatment methods

## Abstract

**Objective:**

To investigate the etiology, clinical manifestations, treatment methods, and prognosis of urethral diverticulum cancer to enhance understanding of this disease.

**Methods:**

This paper reports a case of urethral diverticulum cancer with “hematuria and dysuria” as the main clinical manifestations. The case is discussed in conjunction with relevant literature.

**Results:**

The patient initially presented to the gynecology department due to postmenopausal bleeding but was not diagnosed with the disease. Subsequently, the patient developed hematuria and dysuria and was referred to the urology department. MRI indicated urethral diverticulum-like changes with suspected tumorous lesions. Cystourethroscopic biopsy confirmed urethral diverticulum cancer. The patient underwent urethrectomy, and postoperative pathological diagnosis was poorly differentiated urothelial adenocarcinoma.

**Conclusion:**

Urethral diverticulum cancer is a rare and highly aggressive malignant tumor of the urethra with a poor prognosis. The onset of urethral diverticulum cancer is concealed, and its clinical manifestations are nonspecific and diverse. Preoperative diagnosis is challenging, and imaging studies and cystourethroscopy are the primary preoperative diagnostic methods for urethral diverticulum cancer. Pathology and immunohistochemistry serve as the basis for confirmation of diagnosis. Currently, there is no unified treatment protocol for female urethral diverticulum cancer internationally. For female patients with urethral diverticulum cancer without distant metastasis, surgery remains the primary treatment. For patients with distant metastasis, a combination of surgery, chemotherapy, and radiotherapy may be considered.Genetic testing and targeted immunotherapy offer new approaches for future treatment.

## 1 Background introduction

Urethral diverticular cancer (UDC) is a rare, aggressive tumor that arises within the urethral diverticulum in females. Due to its insidious onset and lack of specific clinical manifestations, UDC is often difficult to detect in its early stages. Consequently, the majority of patients present with advanced disease, leading to poor prognosis. The etiology of UDC remains unknown, and there is no universally accepted standard of treatment on an international scale. This report analyzes a case of UDC treated in our hospital, combined with a review of relevant case literature, to summarize diagnostic and treatment experiences. The aim is to enhance clinicians’ understanding of this disease and thereby provide opportunities for timely patient treatment.

## 2 Case report

The patient is a middle-aged female, 55 years old, admitted to the hospital due to “hematuria and dysuria for one month.”

On April 1st, 2024, the patient consulted a gynecologist due to “menopause for three years and slight vaginal bleeding for 20 days.” During the physical examination, vaginal wall congestion was observed, the cervix was smooth, and a small amount of blood was seen within the vagina. Cervical cancer screening was simultaneously performed, and no intraepithelial neoplasia was found. Color Doppler ultrasonography indicated a 3 mm thickness of the endometrium and a 12x6 mm low-echo nodule in the uterine myometrium, which was considered a uterine fibroid. On April 5th,2024, the patient underwent hysteroscopy, which revealed a normal uterine cavity with no abnormal hyperplasia or occupation. Diagnostic dilation and curettage were performed intraoperatively, and the postoperative pathological report indicated chronic inflammation of the cervical canal and a few fragmented glands in the endometrium. The patient still had bleeding symptoms postoperatively, but the bleeding did not increase. Considering post-hysteroscopy uterine bleeding, no intervention was made. On April 25th, 2024, the patient began experiencing dysuria accompanied by gross hematuria. Urine became thin, and abdominal pressure was needed for urination, with gradually worsening symptoms. On May 1st, 2024, a urethral catheter was indwelled at the community hospital, and a urethral mass was found. Simultaneously, pelvic MRI revealed urethral diverticulum-like changes with tumorous lesions. The patient requested treatment and was admitted to the outpatient clinic on May 9th, 2024, with a diagnosis of “urethral tumor.”

Past Medical History: At 40 years old, the patient underwent a transobturator tape (TVT-O) procedure for “stress urinary incontinence.”

**Figure 1:**
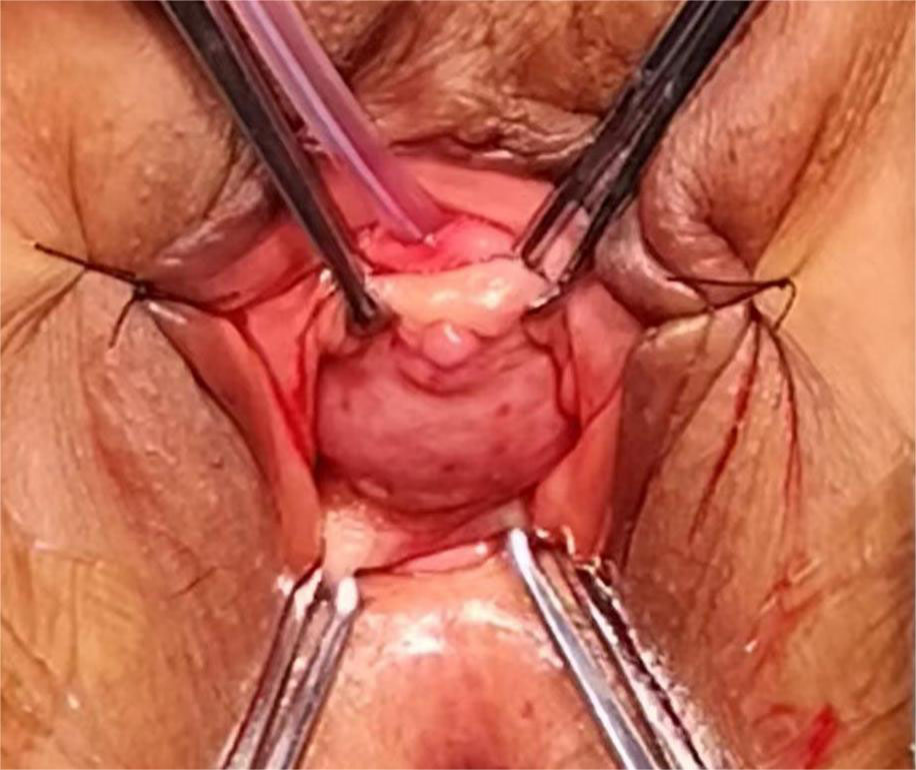
Urethral mass.

**Figure 2:**
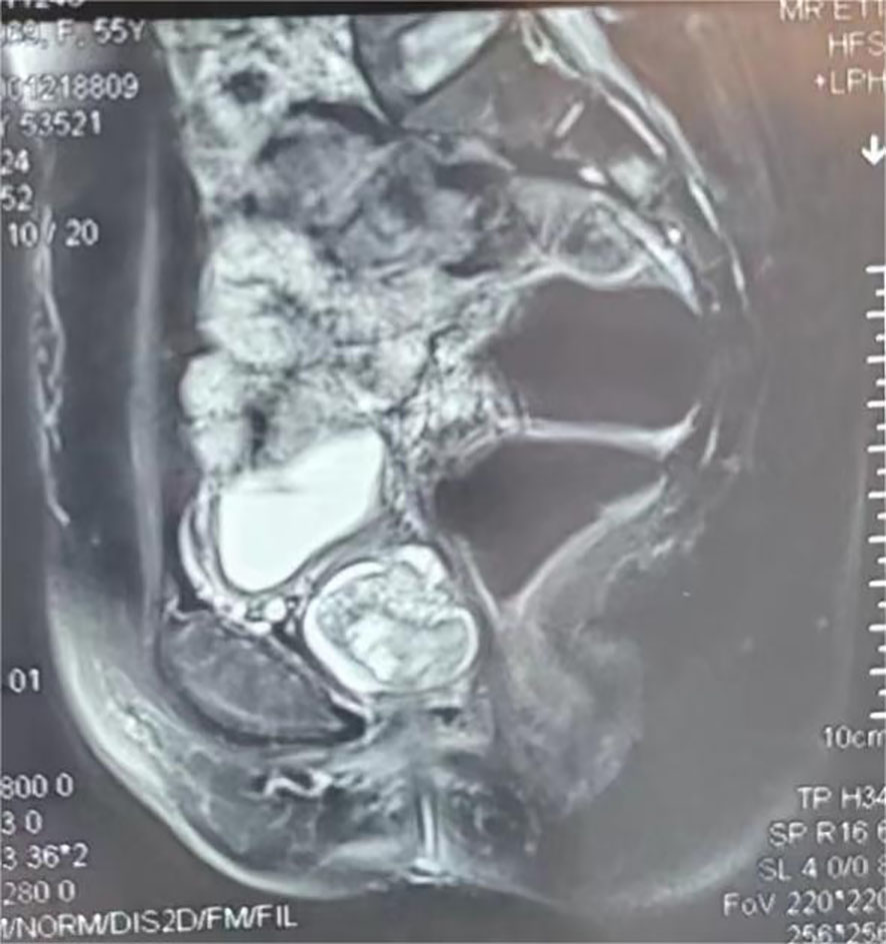
MRI.

Physical Examination upon Admission: Temperature was 36.5°C, pulse rate was 70 beats per minute, blood pressure was 106/78 mmHg, respiratory rate was 20 breaths per minute, height was 1.58 m, and weight was 53 kg. No enlargement of thyroid or cervical lymph nodes was detected. The cardiopulmonary examination was negative, with no pathological murmurs heard. The abdomen was soft, with no tenderness, rebound tenderness, or enlargement of the liver or spleen. There was no edema in the lower extremities. Specialized Physical Examination: The external genitalia were normal, the vagina was patent with a reddish-lush vaginal wall, the cervix was smooth, and no abnormalities were palpable in the uterus or bilateral adnexa. There was no redness, swelling, or secretions at the external urethral orifice. A mass approximately 30x30 mm in size was palpable in the proximal anterior vaginal wall and the lower one-third of the urethra. The mass was slightly soft, bled easily upon touch, had unclear boundaries, and poor mobility (see **Figure 1**). Auxiliary Examination: On May 3rd, 2024, pelvic MRI indicated a cystic shadow in the region below the bladder and the urethra, with a mass lesion inside: urethral diverticulum-like changes with suspected tumorous lesions (see **Figure 2**).

**Figure 3:**
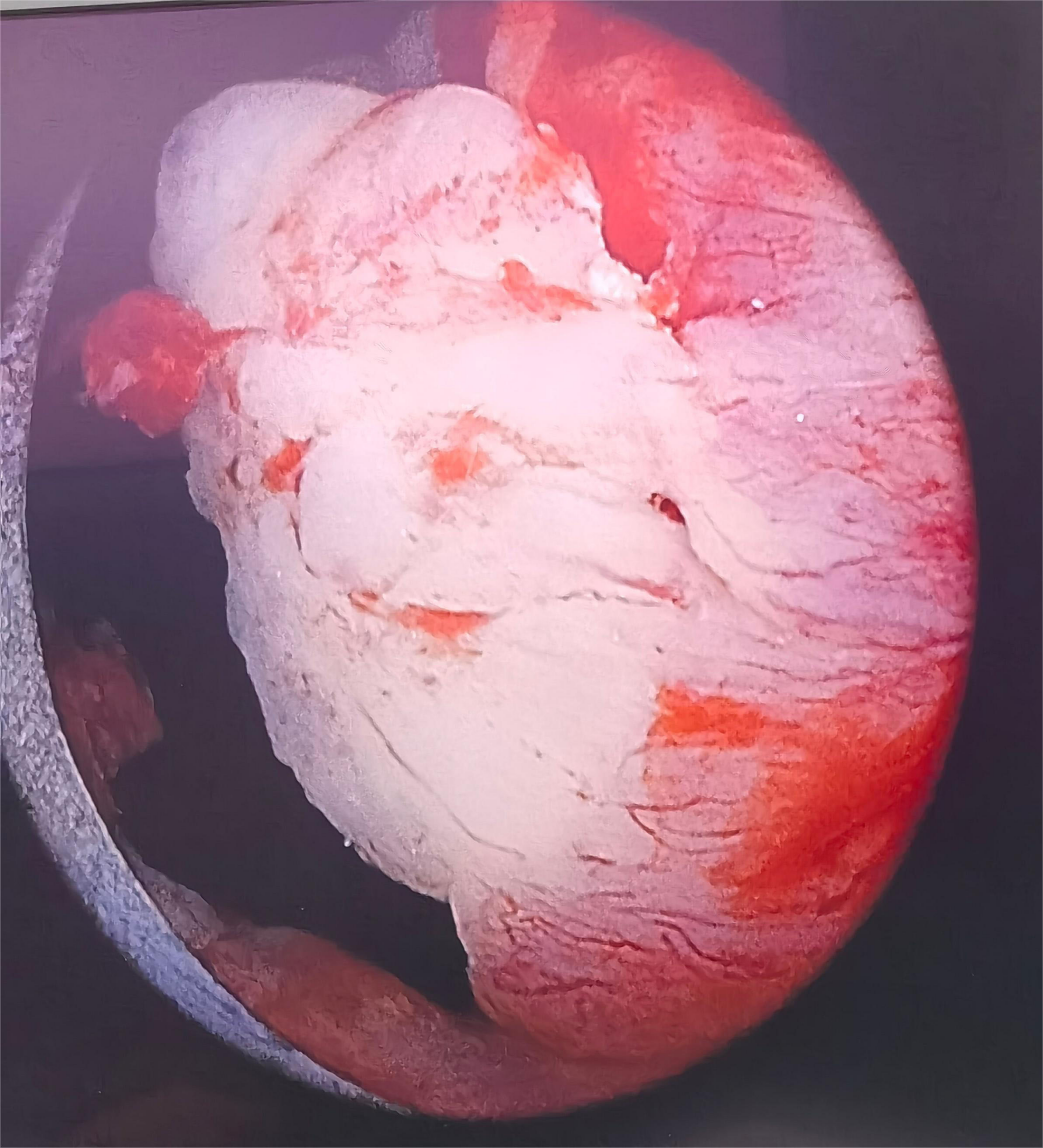
Cystoscopy.

**Figure 4:**
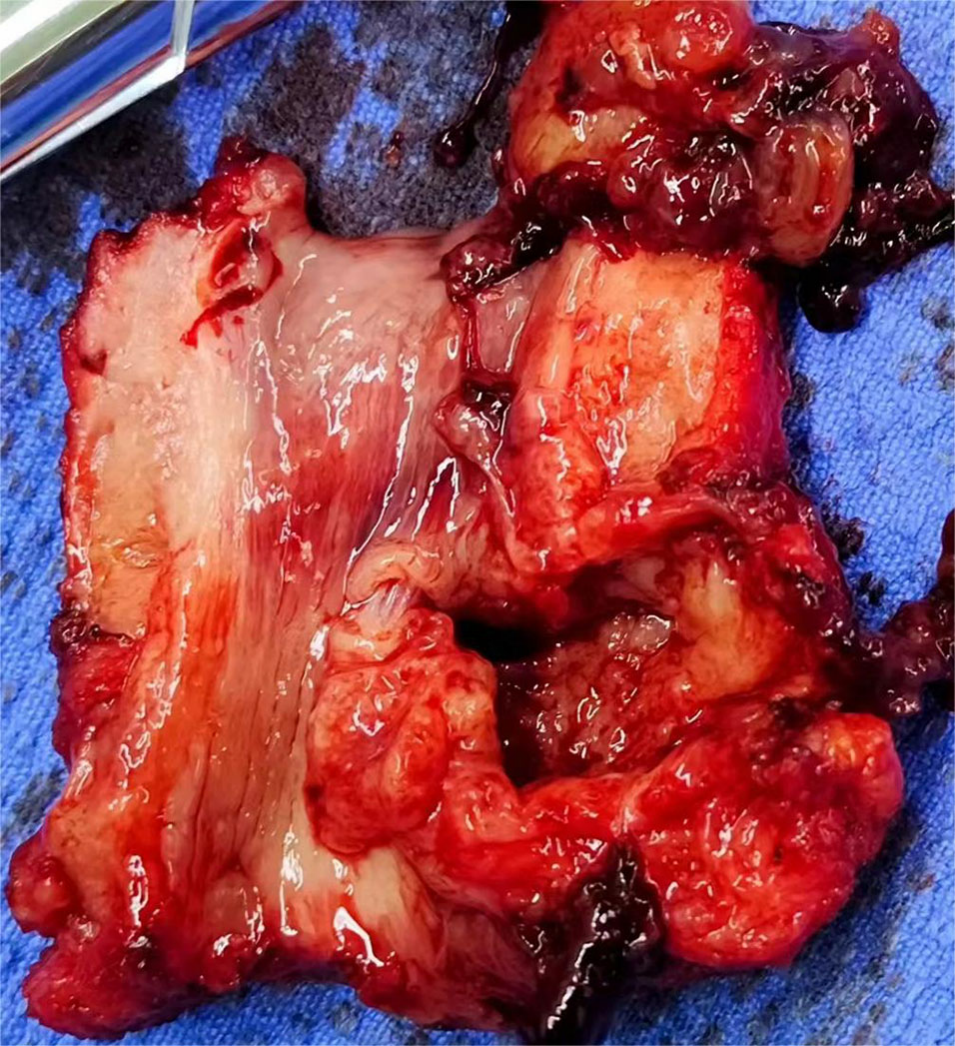
(gross specimen). *CK(Pan), CK8&18, EMA, CK7, and GATA3 all indicate the presence of epithelial components, consistent with the diagnosis of urothelial carcinoma. p63, p53, Ki-67, and HER2 are used to determine the degree of tumor differentiation, malignancy, growth rate, and prognosis. CK5&6 suggests the presence of tumor cells with basal-like features. CD56 indicates the presence of a small number of NK cells or cells with neuroendocrine features in the sample. CK20, CDX2, CgA, and Syn are used to distinguish the origin of the tumor.).

After admission, preoperative examinations were completed and preoperative preparations were carried out. On May 11, 2024, cystoscopy revealed a diverticulum approximately 3x3cm in size located below the left urethra, with cauliflower-like neoplasms inside (see **Figure 3**). Multiple biopsies of the urethral mass were taken intraoperatively, and the postoperative pathological diagnosis indicated a suspected malignant tumor. On the same day, the patient underwent total urethrectomy followed by permanent cystostomy. The resected gross specimen showed the tumor located within the urethral diverticulum (see **Figure 4**). The postoperative pathological diagnosis confirmed it as a poorly differentiated urothelial adenocarcinoma, which invaded the full thickness of the urethral wall and extended into the surrounding connective tissue. No cancer involvement was found at the resection margins of the lesion. Immunohistochemical staining results were as follows:CK(Pan)(+), CK8&18(+), EMA(+), CK7(+), GATA3(partially+), p63(individually ++), p53(-), Ki-67(MIB-1)(+, approximately 70% locally), CK5&6(individually+), CK20(-), HER2(1+), CDX2(-), CgA(-), Syn(-), CD56(mostly -, focally weak+), Rb(+), S100(-), and SOX10(-). Molecular testing showed no mutation in the TERT gene promoter (-). The patient did not receive radiotherapy or chemotherapy and was discharged on May 15, 2024, in improved condition.

One month after the surgery, the patient was followed up at the outpatient clinic, and the bladder stoma was patent with good healing of the urethral stump incision. Follow-up visits at three months and six months postoperatively both showed no recurrence of the tumor.

## 3 Discussion

GM Grimsby (1) reported in the literature that the incidence of female urethral diverticulum is approximately 1.85% to 6%, and urethral diverticulum cancer originating from within the urethral diverticulum accounts for about 5% of urethral diverticula and 0.01‰ of all female genitourinary malignancies (2). O’Connor (3) et al. summarized 126 cases of UDC from domestic and foreign literature and concluded that the age of onset of urethral diverticulum cancer ranges from 14 to 81 years, with a higher incidence in older age groups and an average age of 53 years. The pathogenesis of urethral diverticulum cancer is currently unclear and is mainly classified into congenital and acquired types, with most scholars supporting the formation of acquired factors. They believe (4) that urethral diverticulum cancer results from exogenous trauma (recurrent infections of periurethral glands, vaginal delivery trauma, previous vaginal or urethral surgery) leading to diverticular changes in the urethra. This results in obstructed drainage of the paradiverticular glands, causing expansion and infection of the surrounding glands. Under the stimulation of various chronic infections, the glandular epithelium surrounding the diverticulum undergoes carcinogenic transformation. In our report, both cystoscopy and MRI identified urethral diverticula, which also suggests that urethral diverticulum cancer may originate from the glands surrounding the urethral diverticulum.

UDC predominantly occurs in the mid or distal urethra, with 58.5% of cases originating from this location (5). Histologically, UDC is classified as adenocarcinoma (56%), transitional cell carcinoma (29%), and squamous cell carcinoma (15%) (6). Adenocarcinoma is further subdivided into mucinous and clear cell subtypes. The clinical manifestations of UDC lack specificity, and approximately 20% of patients may have no early clinical symptoms. The main clinical manifestations of urethral diverticulum cancer (3) include urethral bleeding/hematuria (55%), urethral obstruction/voiding dysfunction (16%), intraurethral mass (13%), recurrent urinary tract infections (13%), dyspareunia (7%), localized urethral pain (6%), and post-void dribbling/incontinence (3%). During physical examination, 85% of patients may have a painless mass palpable on the anterior vaginal wall. The dissemination of UDC typically involves direct infiltration and lymphatic spread. Direct infiltration includes proximal invasion of the bladder neck and distal invasion of the vestibule, labia, and vagina, potentially leading to the formation of urethrovaginal fistulas in later stages. Distant metastases can involve pelvic and inguinal lymph nodes.

Due to the nonspecific and diverse clinical manifestations and signs of UDC, preoperative diagnosis of the disease is extremely challenging. Physical examination is necessary to differentiate UDC from cystocele, paravaginal cysts, paraovarian cysts, endometriosis, urethral cancer, and vaginal cancer. The definitive diagnosis of UDC relies on postoperative pathology and immunohistochemistry, but imaging studies and cystourethroscopy can provide guidance for diagnosis and surgical approaches. Urine cytology (7) can serve as a useful initial screening test with a positive rate of 85% in UDC, but it cannot determine the histological subtype of the tumor. Ultrasonography is the preferred non-invasive examination and is sensitive for detecting UDC. CT scanning is also an important diagnostic tool for UDC and assessing clinical staging. MRI is considered a highly sensitive and specific imaging modality for diagnosing urethral diverticula. MRI can provide detailed information on the location, number, size, morphology, relationship with the urethra, and local invasion scope of UDC. Cystourethroscopy, as a routine method for obtaining a definitive preoperative diagnosis, allows for more direct observation of the tumor’s location, size, morphology, invasion scope, and involvement of the bladder, as well as determining the ease of complete resection of the lesion during surgery. However, there are patients with UDC who have normal cystourethroscopic findings and may require ultrasound-guided biopsy of the urethral mass. Immunohistochemical markers (8, 9) are helpful for accurately diagnosing the origin and histological classification of UDC, assessing prognosis, and providing treatment plans for postoperative chemotherapy.

Currently, the primary treatment for UDC is surgical intervention. Patel (10) studied the prognostic factors in patients with UDC and concluded that surgery can improve patient survival rates and reduce cancer recurrence. However, there is no standard surgical protocol for UDC, and surgical treatment varies depending on the stage and location of the tumor (11). For smaller, superficial UDC tumors located in the anterior urethra, partial urethrectomy or tumor excision may be selected, provided that a negative surgical margin is ensured. For posterior urethral diverticulum cancer, anterior exenteration is generally required. Radical urethrectomy is indicated for larger tumors or tumors involving the entire urethra. Currently, prophylactic and diagnostic lymph node dissection is not recommended; lymph node dissection is only recommended for patients with UDC who have inguinal or pelvic lymph node metastasis without distant metastasis. For advanced UDC, a multidisciplinary comprehensive treatment approach combining surgery, radiotherapy, and chemotherapy is often adopted. Seballos (12) et al. suggested adjuvant chemotherapy when UDC involves the bladder neck. Related reports indicate (11, 13) that combination therapy with platinum-based chemotherapy on the basis of surgery can reduce tumor size and recurrence in patients with UDC. A recent study showed (14) that the addition of brachytherapy to external beam radiation therapy can reduce the risk of local recurrence by 4.2 times. With the development of tumor genetic testing technology, targeted drug therapy with HER2 inhibitors and biological immunotherapy with NK cells now provide new directions and references for precision chemotherapy in UDC. The prognosis of UDC varies among different histological types. Shalev (15) reviewed data from 97 patients with UDC and found that the cancer-free survival rates for adenocarcinoma, transitional cell carcinoma, and squamous cell carcinoma were 54%, 43%, and 22%, respectively. Masakazu Gonda (16) et al. reported that the main factors affecting the prognosis of UDC include age, race, clinical stage, tumor location, tumor volume, pathological type, and treatment. Among them, the stage of the primary tumor, the presence of regional lymph node involvement, and the location of the disease (proximal vs. distal urethra) are all independent risk factors for survival (17). Overall, the prognosis for patients with UDC is poor, with a low five-year survival rate.

In summary, UDC is an extremely rare disease. Due to its insidious onset, nonspecific clinical manifestations, and unclear physical signs, it is prone to missed diagnosis or delayed medical attention. Physical examination is crucial in clinical diagnosis, as it can detect most cases of UDC in advance and improve the positive screening rate. UDC lacks specific laboratory indicators, and imaging studies can serve as an initial screening tool, but definitive diagnosis requires cystourethroscopy and pathological results. Currently, the etiology of UDC is unclear, and there is no consensus on its treatment plan internationally. Several small case studies have shown that surgical treatment is the preferred and effective approach for small, distal, and early-stage UDC; for metastatic UDC, a comprehensive treatment plan combining surgery, radiotherapy, and chemotherapy is required. Targeted therapy and immunotherapy are goals for future medical exploration. The overall prognosis of UDC is poor, and the disease is prone to recurrence. Therefore, patients need regular follow-up after discharge to intervene in disease progression in a timely manner and help achieve satisfactory prognosis.

## Data Availability

The original contributions presented in the study are included in the article/supplementary material, further inquiries can be directed to the corresponding author.
